# The development of the status of Druze women in the 21st century

**DOI:** 10.3389/fsoc.2023.1206494

**Published:** 2023-09-21

**Authors:** Janan Faraj Falah

**Affiliations:** Western Galilee College, Acre, Israel

**Keywords:** Druze, society changes, women status, education, new society

## Abstract

This article analyzes the social, personal, and religious reasons for the change in the status of Druze women in Israel. In the 1970s, the first wave of female Druze teachers began when a class intended only for women was opened. In the last 20 years, there has been a fundamental change in the status of women, followed by a change in the status of women in Israel in general. This change resulted in an increase in the percentage of female Druze students compared with male Druze students. According to data from the Israeli Central Bureau of Statistics, in 2020, 68% of all Druze B. Undergraduatestudents and 64.8% of M.A students were Druze women. This increase has affected the various professions in which Druze women work. This article will also discuss the effect of the change in the status of Druze women on Druze society in general.

## 1. Chapter 1. The Druze community

### 1.1. The social characteristics of the Druze community

The Druze are a heterodoxy community. It was founded in the 11th century in Fatimid Egypt, during the rule of the Sixth Fatimid Caliph Al-Hakim bi-Amr Allah, between the years 996 to 1021. Toward the end of his reign, the Druze sect began to form.

The Druze doctrine is greatly influenced by the Ismaili doctrine (Hazran, 2007; Hassan, 2011). After more than 1,000 years since the beginning of the Druze religion, today, the Druze live in four main countries in the Middle East: Syria (~700.00 Druze), Lebanon (~215,000 people), Jordan (~30,000 people) and in Israel (~147,000 Druze). There are small communities in other countries as well, mainly in North and South America, Australia, and South Africa.

According to the Central Bureau of Statistics, by the end of 2020, the Druze community in Israel numbered ~147,000 people, which is 1.6% of the total population in the country and 7.6% of the Arab population in Israel. Ninety-eight percent of the Druze in Israel live in 19 villages (17 villages in the Northern District and two in the Haifa District). The total fertility rate of the Druze women is relatively poor in other sectors and stands at 2.02 children. In 2019, there were ~39,000 Druze households, and the age of first marriage is 28.7 for men and 25 for women. There is a large gap between the participation rate of men and women in the labor market; Druze men make up 61.2% of the workforce and women stand at only 34.5% (CBS, 2021).

The Druze in Israel have maintained good relations with the Jewish population since the beginning of the Jewish State and have supported the establishment of the state and made an ally with it. In 1957, the Druze Community was recognized as “an independent religious community” by the Minister of Religions, and in October 1961 “the Religious Council” (of three members) was recognized as the main religious authority of the Druze community. This process was completed on December 25, 1962, with the Knesset's approval of the Druze Court Law as the sole religious authority for Druze affairs (Falah, 2000). Another important religious aspect is the distinction between religious (the Ok'al) and secular (Jo'hal), which is emphasized in terms of clothing, customs, and lifestyle (Hassan, 2011). The religious sage is the leader of the community; thus, their opinion is also considered in matters that are not exclusively related to religion (Shtrukman, 2010). In addition, the Druze religion forbids polygamy, women and men are religiously equal, and women are entitled to fulfill any religious role as well as being equal in matters of inheritance (Farraj Falah, 2016).

### 1.2. Social characteristics

The Druze community is conservative and characterized by a traditional way of life and a patriarchal structure (Amrani, 2010; Halabi and Shamai, 2016; Farraj Falah, 2017), while constantly caring about preserving the key values of religion, tradition, independence, and the connection to the land (Falah, 2000; Hassan, 2011). The Druze family is characterized by a clear hierarchy, and the father has absolute rule over his extended family, i.e., his wife, sons, unmarried daughters, brides, and their offspring. Everyone is required to obey and respect the elders of the family; furthermore, family goals overcome personal ones (Amrani, 2010; Hassan, 2011; Farraj Falah, 2016). The unequal inferior status of Druze women is emphasized within the family as they are required to take care of the housework and the children, maintain a modest appearance, avoid contact with strangers, and obey their husbands, yet, at the same time, their status is not static and improves over time as the Druze woman grows older, mainly due to her married sons who build their house nearby (on the same property), thus contributing to the expansion of the extended family. Her understanding and involvement in her son's marriage, in the management of family affairs, and the obedience of her daughters-in-law strengthen her position within the family unit (Wiener Levi, 2005a,b; Barkat, 2016).

### 1.3. A community in a process of change

Yashiv and Klinet-Kassir (2018) explains the expansion of secular education as part of modernization and media development processes that led to the undermining of the patriarchal religious structure. The Druze interact with other communities regularly, particularly the younger generations of Druze males with the dominant Jewish sector during their military service, which led to modernization and deepened the process. This means that beyond the identity problem, the Druze Community in Israel has experienced a continuous modification and assimilation to Western culture for the past three decades, and as a result, the family structure has transformed from a traditional collective extended unit to a modern Western one, with limited consideration of the extended family (Shtrukman, 2010).

One of the characteristics of Druze youth in the post-modern age is the desire for independence; this desire also weakens the extended family structure and strengthens the nuclear family pattern of two parents living with their unmarried children. Various factors affect this transition in the family pattern, which contributes to a wider transition within the social structure of the entire Druze Community; for example, the establishment of new neighborhoods for veterans who choose to live outside their family territory. The independence of the younger generation along with the increase in the level of education undermined the authority of the traditional leadership, which is more religious and older (Farraj Falah, 2005). In addition, the exposure of young Druze men during their military service to a lifestyle that is fundamentally different from life in the Druze village, as well as the one-way relationship with the city, manifested in frequent visits from the village to the city to study, work, or have fun and also exposed the younger generation to Western culture (Farraj Falah, 2005; Shtrukman, 2010). The social change is further characterized by an occupational transition from agriculture at the beginning of the state within the boundaries of the village to public servants in the defense forces, outside the village. The work outside the village also includes the self-employed, thus contributing greatly to changing traditional perceptions regarding the land and extended family (Shtrukman, 2010).

A dominant factor in the Community's alteration is the change in the status of the Druze women. Women began to acquire a broader education and work in the profession they learned outside the village. This factor reinforces the change. Today, in most Druze Communities, against the older religious leadership, a new generation is emerging: a generation that holds senior public positions in the government and the security arms.

These changes cause tension between the religious and the secular and intensify the inter-sectarian conflict on the issue of “Druze who abandoned the basic community values” (Falah, 2000; Shtrukman, 2010).

## 2. Chapter 2. The development of the Druze woman from the 1980s to today

This section presents data on the development of Druze women in the last decade in two main aspects of life: education and employment.

### 2.1. Education among Druze women over the last decade

As mentioned above, the appeal of academic studies to Druze women began in the early 1990s.

The figures below present the percentage of Druze in the Israeli academy in general and among Druze women.

**Figure 1:**
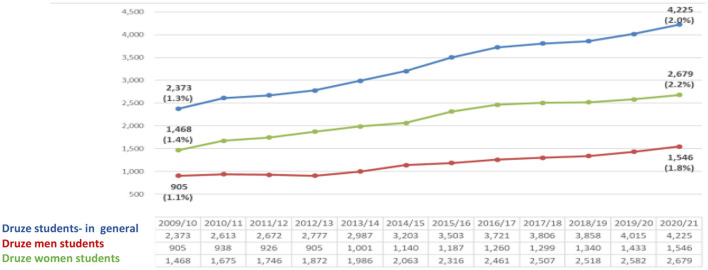
Druze B.A. students at CBS (2021) (Veniger, 2021).

The data proves that the number of male and female Druze B.A. students studies has increased. Yet, their proportion in the general population in the last year (an increase of 2%) is higher than the rate of Druze in the general population of Israel (1.6%). However, the rate of Druze female students is higher (2.2% rise) than male Druze (1.8%). Thus, female Druze students made up ~63% of all Druze undergraduate students (Figure 1).

**Figure 2:**
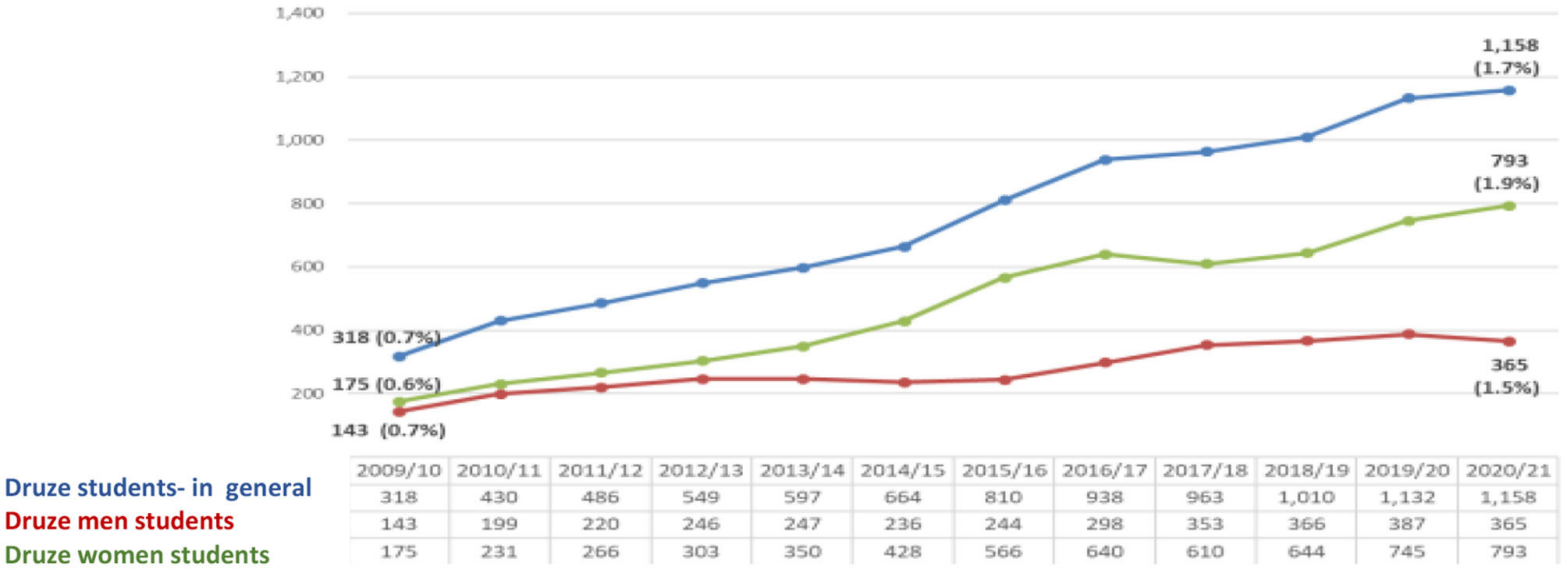
Druze M.A. students at CBS (2021) (Veniger, 2021).

The data in the figure demonstrates an increase in the number of Druze students (men and women) studying for a master's degree in Israel. The participation rate of the Druze students in general last year in higher education (1.7%) is higher than the rate of Druze in the general population in Israel (1.6%) and the rate of Druze women in the last year is higher (1.9%) than the rate of Druze male students (1.5%), with women accounting for 68% of all M.A. Druze graduate students (Figure 2).

**Figure 3:**
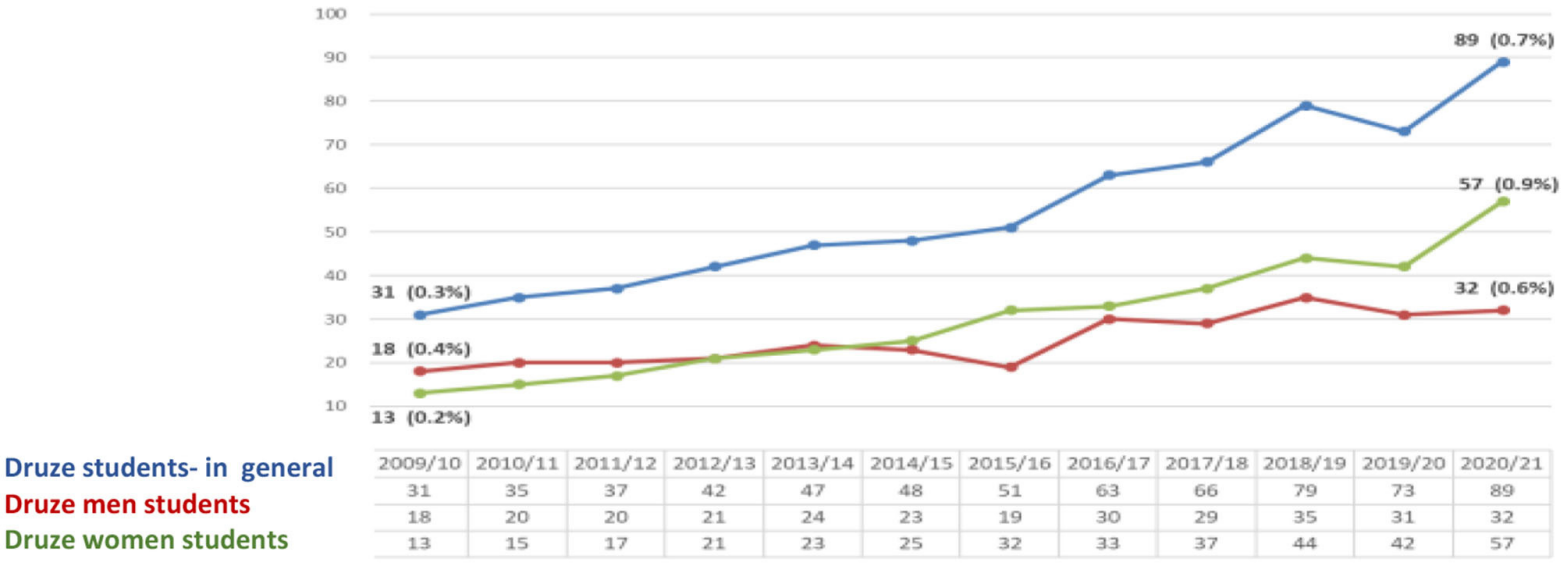
Druze Ph.D. students at CBS (2021) (Veniger, 2021).

The data prove that the number of Druze students in general (men and women) studying for a Ph.D. has increased. Despite the increase of Druze students over the years (0/7%), the proportion of Druze in the general population in Israel in the last year is still lower (1.6%); however, the proportion of female Druze students is higher (0.9%) than the corresponding rate among Druze men (0.6%) and stands at 64% of all Druze postgraduate students (Figure 3).

**Figure 4:**
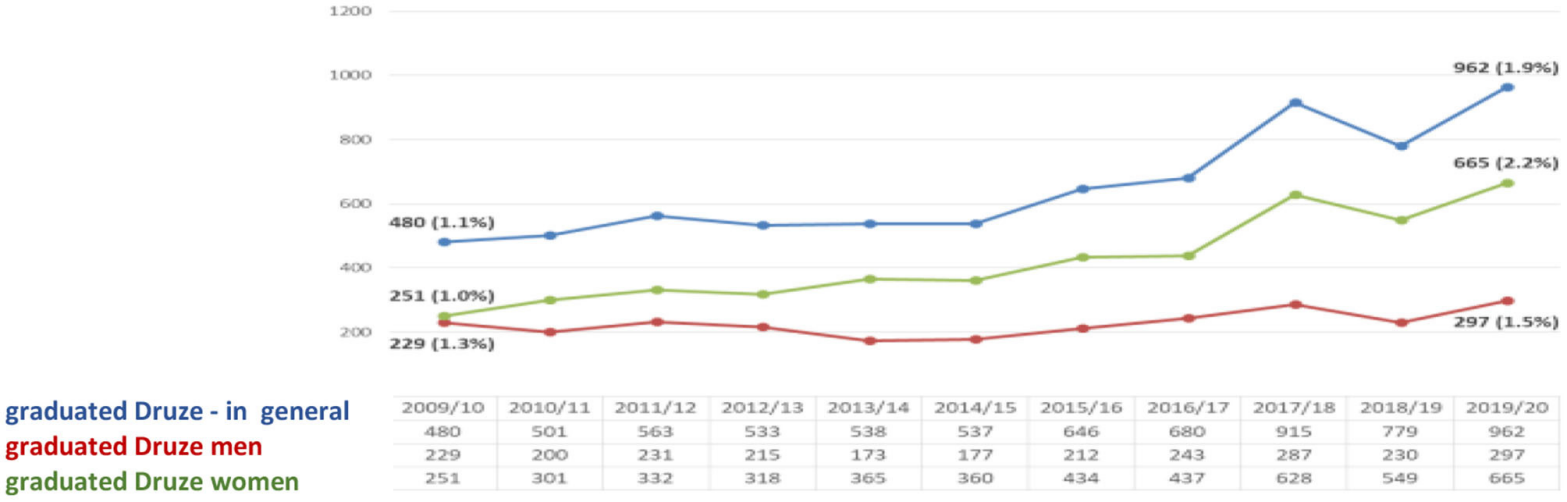
Druze B.A. graduates at CBS (2021) (Veniger, 2021).

The data in this figure illustrate that, over the years, the number of graduated B.A. Druze has increased. Their share (1.9%) is higher than the share of Druze in the general population of Israel (1.6%). In the last year, the rate of graduated B.A. Druze women (2.2%) is higher than the rate of male graduated Druze (1.5%) (Figure 4).

**Figure 5:**
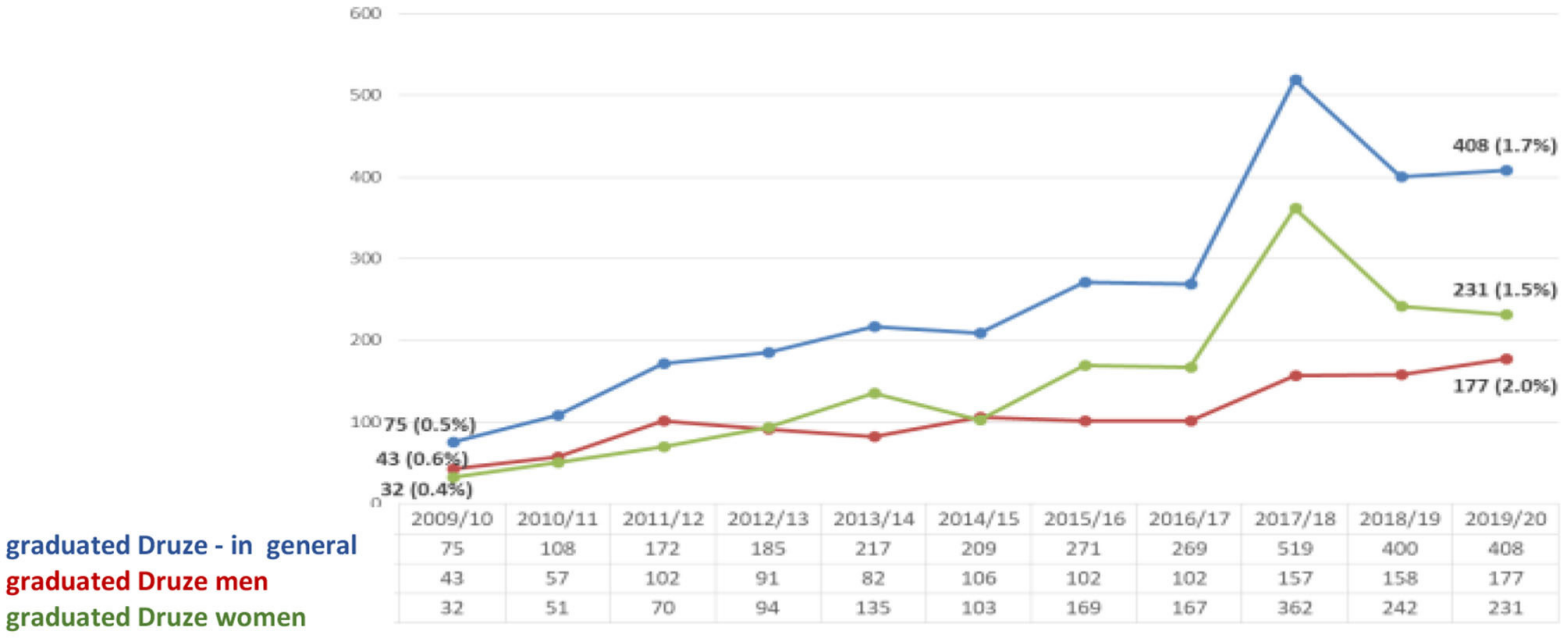
Druze M.A graduates at CBS (2021) (Veniger, 2021).

The figure proves that the number of Druze M.A. graduates has increased. In addition, their share (1.7%) is slightly higher than the share of Druze in the Israeli general population (1.6%). In the last year, the rate of female Druze M.A. graduates (2.2%) is higher than the corresponding rate among Druze men (1.5%) and stands at 57% of all Druze with a master's degree (Figure 5).

**Figure 6:**
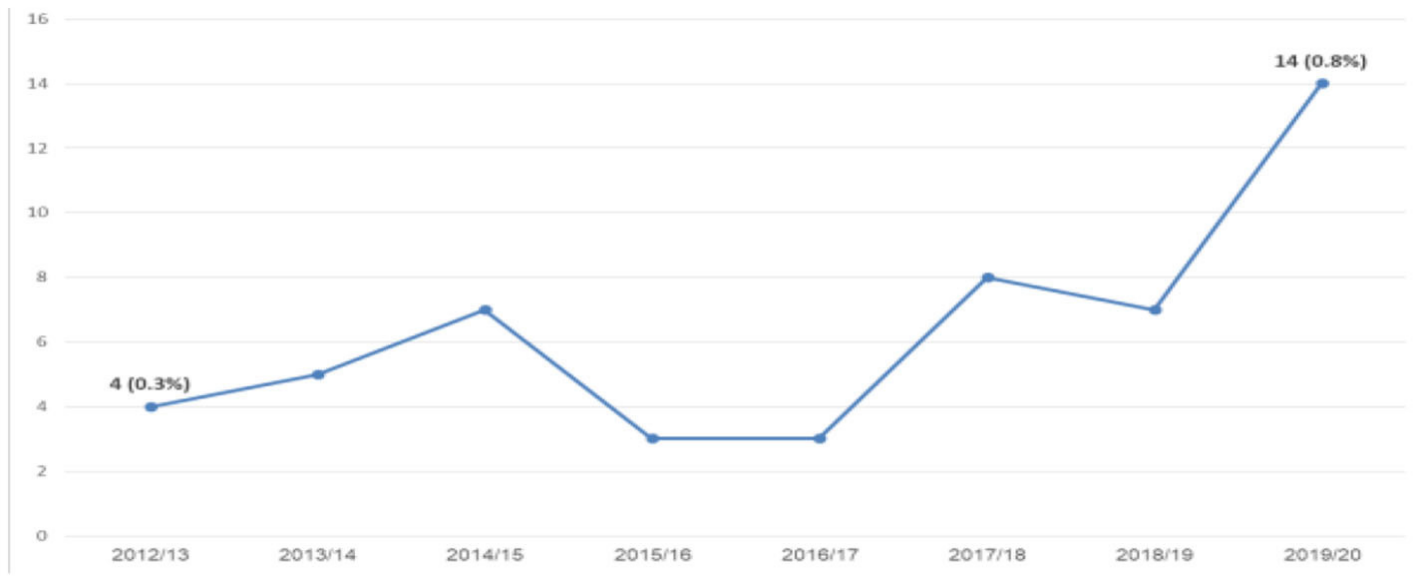
Druze Ph.D. graduates at CBS (2021) (Veniger, 2021).

The Druze Ph.D. graduate data demonstrate a significant increase. Despite the increase that has occurred over the years, their rate (0/8%) is still lower than the share of the Druze in the general Israeli population (1.6%) (Figure 6).

The data highlight the gap between the percentage of graduated Druze (15.1%), graduated Jews (27.0%), and graduated Christian Arabs (22.6%). However, their percentage in the Muslim Arab population (9.7%) is higher.

The rate of graduated female Druze (19.4%) is higher than the proportion of graduated Druze men (11%).

**Figure 7:**
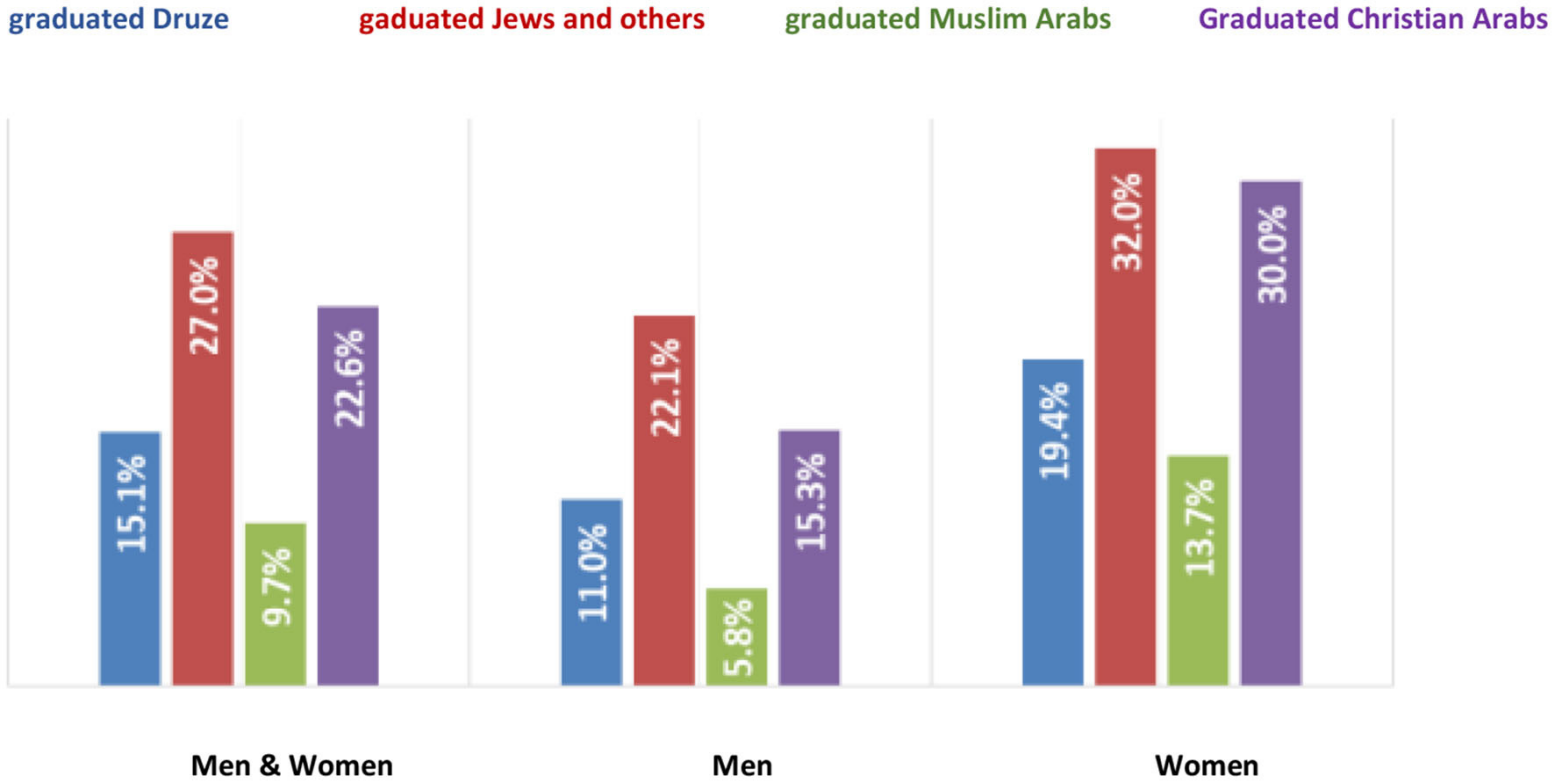
The percentage of Israeli degree holders between the ages of 24 and 55 years old, according to the CBS (2021) (Veniger, 2021).

In light of the numbers, it can be concluded that during the second decade of the 21st century, the acquisition of higher education by Druze women accelerated dramatically. Moreover, the percentage of Druze women in the academy was higher than the percentage of Druze men (Figure 7).

### 2.2. The development of Druze women's participation in labor

It has already been mentioned that Druze women made great progress in the field of education, a factor that led to an improvement in working conditions and employment rates, although this process was more limited. This section discusses the participation rates of Druze women in the labor force over the past 25 years.

**Figure 8:**
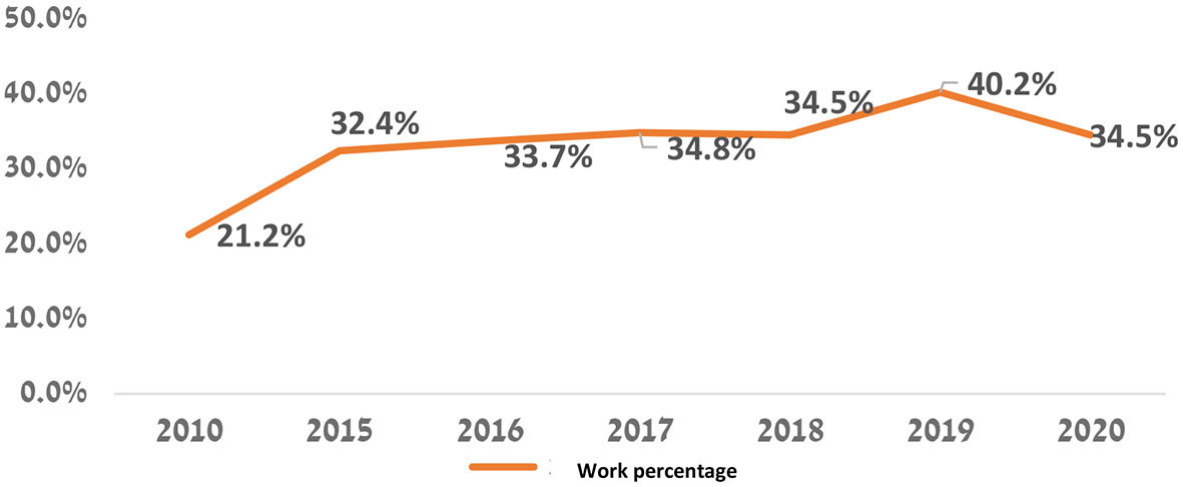
Percentage of Druze women over the age of 15 in the workforce, according to the CBS, 2010, CBS, 2020 (Mezrahy and Elyaho, 2015).

The graph proves that the participation rate of women by all religions in the Israeli labor force has increased over the years. There is a significant increase in the participation rate among Druze women, i.e., in 2010, the participation rate of Druze women was only 21.2%, and 10 years later in 2020, it was 34.5%. At the same time, their share in the labor force, among Jewish and Christian women, remains much lower. However, CBS data in the year 2019 show that the employment rate of Druze was 40.2%. The decrease between these years is related to the coronavirus outbreak in 2020 (Figure 8).

The issue of the participation of academic women in the labor force is important and central to the study of the development of Druze women.

Acquiring higher education enabled women to participate in the labor force, especially in cases of the Druze villages where the employment environment is poor and lacks appropriate industrial infrastructure, shopping centers, etc.

**Figure 9:**
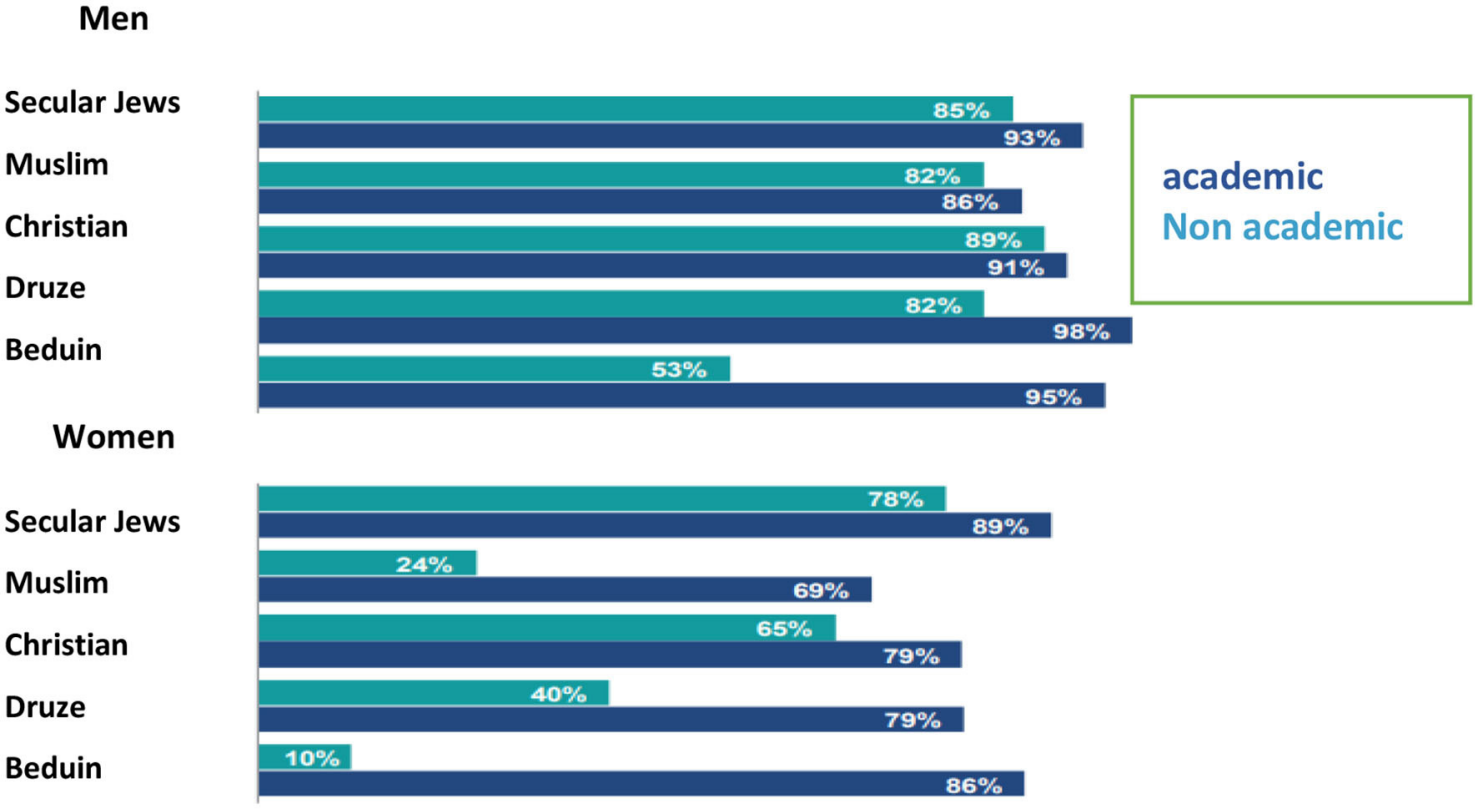
Employment rates (ages 25–34) by education and religion, 2013–2015 (CBS, 2015; Mezrahy and Elyaho, 2015).

The percentage of academic employment rates among Druze women (79%) is twice as high as the percentage of employed non-academic women (40%). This gap merely illustrates the importance of higher education for participating in the workforce (Figure 9).

**Figure 10:**
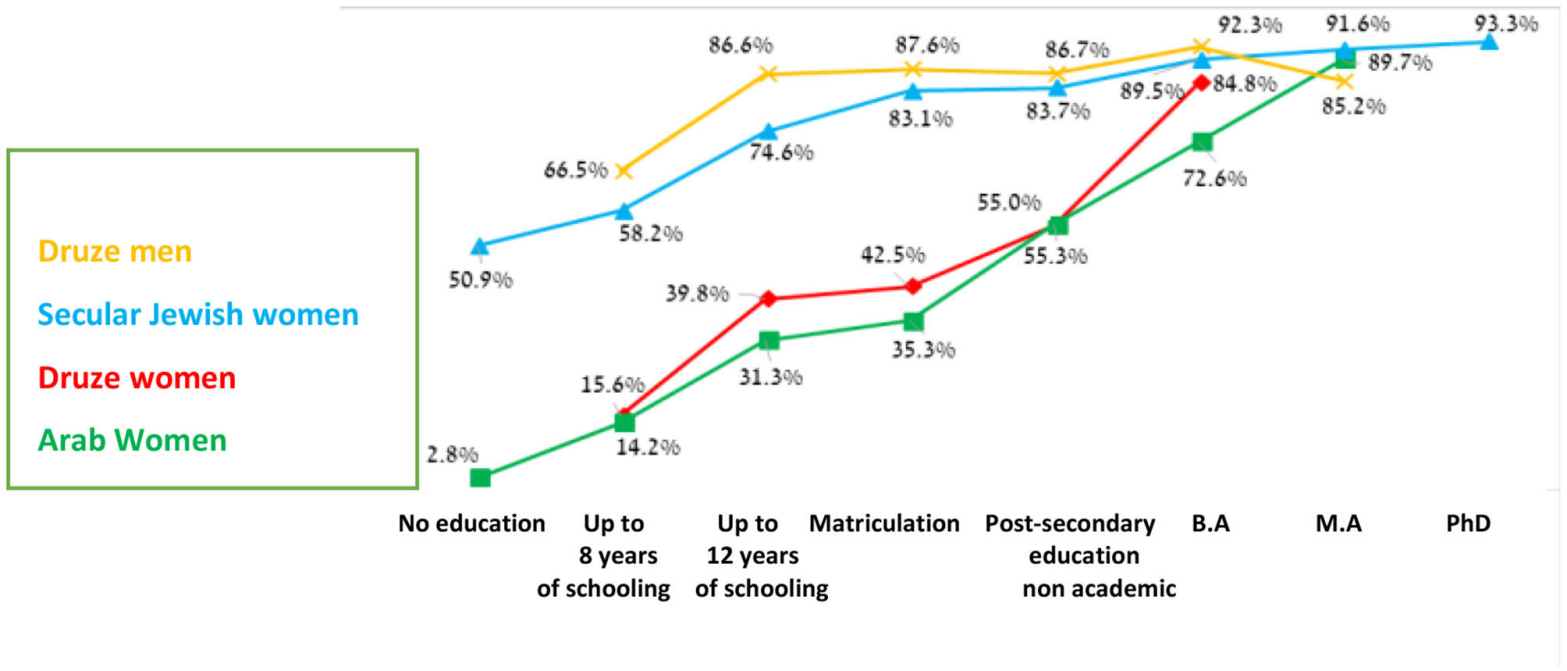
The labor force by education (ages 25–64) (CBS, 2015; Mezrahy and Elyaho, 2015).

The graph demonstrates that the contribution of education to employment is significant. In general, the higher the level of education, the higher the labor force participation rate. The rate of participation among Druze women in the workforce increases greatly with the level of education, especially with an academic degree; if women hold matriculation the participation rate is 42.5%, which increases to 84.8% with a B.A. (Figure 10).

**Figure 11:**
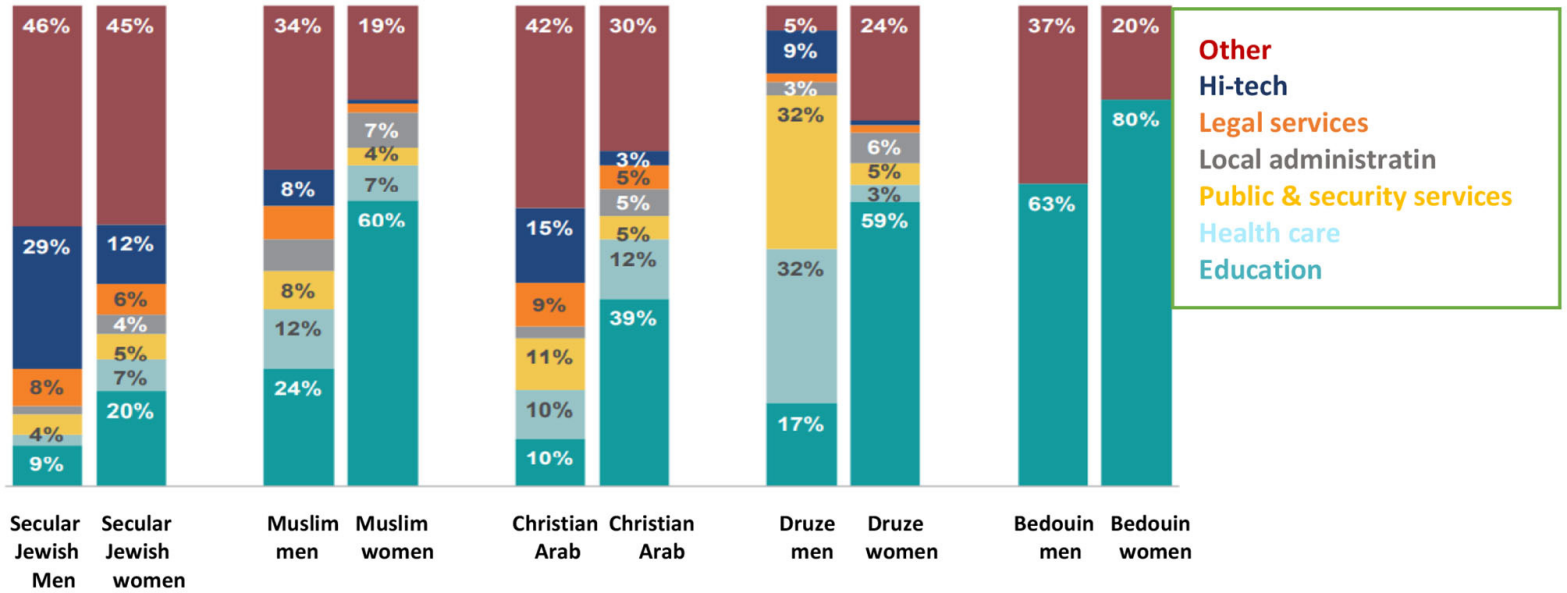
Employment distribution among the highly educated between the ages of 25 and 35, 2013 (CBS, 2015; Mezrahy and Elyaho, 2015).

Examination of the distribution of the labor force by branches among female Druze academics exemplifies that 60% of the women work in the field of education 59%, 24% work in various branches, and a very small percentage work in healthcare (3%), local administration (6%), and public service (5%) (Figure 11).

### 2.3. Analysis of the development of Druze women in general and in the second decade of the third millennium in particular

#### 2.3.1. The numbers 'speak for themselves'

The data presented above indicate two tendencies—increase and growth. The first is achieved by higher education and the second is realized by entering the Israeli labor market. In the higher education, the tendency is high, for example, in the year 2010 the percentage of Druze women among all undergraduate students in Israel was (1.4%)−468 female students, while in 2020 the proportion of Druze women increased to 2.2% (2,679 female students) of all undergraduate female students. As part of the master's degree studies, If in the year 2010, the number of female Druze students was 0.6% (175 female students) of all M.A graduate students, in 2020 their rate is already 1.9% (793 female Druze students) of all students. With regard to Ph.D. studies, it appears that in 2010 the percentage of Druze female students was 0.2% (13) of all Ph.D. graduate students, while in the year 2020, the percentage stands at 0.9% (57). At the same time, there is an increasing trend in the proportion of Druze women participating in the Israeli labor force. In 2010, their participation rate was 21.2%, while in 2015 their rate was 32.4%. The constant moderate tendency is increasing in 2019, and the percentage of working Druze women stands at 40.2%. The working rates dropped in 2020 to 34.5%.

This is genuine progress for Druze women, followed by a rapid and continual improvement in education and an increase in employment rates, although for education, this process is more gradual. In addition, many of the women continue to study B.ED (Bachelor in Education) and seek work in this field, even though the workforce in this field is saturated. The data in Figure 10 prove that a higher level of education leads to greater participation in the labor force; for instance, among women with the most matriculation diplomas, the employment rate is 42.5%, while for women with a bachelor's degree, the employment rate rises to 84.8%. This strengthens the argument that the labor force participation rate among Druze women increases significantly by the level of education, particularly with academic education.

An in-depth examination of the job profile among academic workers proves that 60% of Druze with higher education work in the field of education, another 24% work in various other branches, and a very low percentage of women work in healthcare, local administration, and other areas. From all of the above, it can be concluded that academic Druze women tend to work in teaching, a profession that enables them to balance work and family and maintain the cultural norms and customs of the Druze society. Druze women fully/partially fulfill their aspirations, both in their careers and professional development and caring for their family and children, as well as maintaining the religious norms and social values of the Druze society. To analyze the unique factors that contribute to women's progress in these two aspects, first one must examine which of the two areas affects the other. That is, does higher education encourage work, or vice versa?

The hypothesis is that the two issues, the acquisition of higher education and employment, are interrelated.

#### 2.3.2. Analysis of the impact of the external environment on the internal environment

*[The* ***PEST*** *(Political, Economic, Social, and Technology) model analyzes the external environment of the community in* *political terms*.

*The* **SWOT** *model (strengths, weaknesses, opportunities, and threats) attempts to understand and map the internal environment of the community. The model maps the strengths, weaknesses, opportunities, and threats.]*

The proposed analysis includes two elements. The first is time, a specific timeline. The second element is the factors that affected the community, both in external environmental terms in which the community lives and by internal environmental terms, within the community, and how it was affected by the changes in the external environment.

The analysis is a business-based platform, in which the organization formulates a strategy—the way the organization operates in the external and internal context (the environment) and the challenges and complexities, setting goals, deciding how to act, and methods of action and implementation. The strategy aims to focus on a plan that will maximize the chances of exercising goals and being successful over time. In a world where the pace of change is ever-increasing, it is important to use the right strategy and update and adjust to the changing reality while maintaining a clear mind over time (Samuel, 2005; Johnson and Scholes, 2008).

One way to build a strategy is to analyze the external environment and its effects on the organization and then analyze the internal environment and how it adapts to external environmental changes (Porter, 1998; Samuel, 2005). In the case of the Druze community, the assumption is that the community is a type of organization, and hence, it exists within an external environment and has an internal environment. Therefore, the analysis of the impact of the external environment will be examined by the PEST (Political, Economic, Social, and Technology) model. This model analyzes the external environment of the community in political terms; the political environment has a great impact on the entire function of the environment. The economic environment has a great impact on the economic behavior of the environmental functions. The social environment is defined by the social and cultural influences on subcultures (ethnicity and sectors). The technological environment is defined by the effect of technologies on the openness of the environmental functions. The external environment of the community affects all the functions in the same environment in which the community takes place. As the Druze community cannot affect the external environment, the assumption is that the community will map the changes and adjust itself to them.

The analysis of the internal environment of the Druze Community is based on the SWOT model (strengths, weaknesses, opportunities, and threats), to understand and map the internal environment of the community. The model maps the strengths, weaknesses, opportunities, and threats of the community at a specific time. The assumption is that the strengths and weaknesses are its assets and part of the structure of the Druze community, whereas the opportunities and threats are the projection of the external environmental dynamics on the community. An analysis of the two models over a selected period will be used to reconstruct the strategy of the Druze community.

**1. The eighth decade of the 20th century:**

**A. PEST analysis:**

The political environment: Political stability. Until 1983, the Likud Party was the ruling party. In 1984, a national unity government was formed with a broad coalition of the two major parties in Israel, the Likud Party and the Labor Party (Ma'rach), based on a rotation agreement headed by the prime minister. In the 1988 elections, the Likud won the elections by thousands of votes. Shamir, the head of the Likud, established a national unity government without a rotation agreement. In 1982, during the Lebanon War, Operation Peace for Galilee began with the aim of protecting the Israeli settlements in the north from terrorist attacks, mainly by the Palestinian organizations in southern Lebanon. Israel occupied parts of Lebanon and the IDF remained on Lebanese soil for a decade. In 1987, the first intifada took place, during which the Arab residents of Judea, Samaria, and Gaza revolted against the Israeli government.

Economic environment: In those years, Israel experienced soaring inflation, with a record of 444.9% a year. Although toward the end of the decade, the inflation rate dropped to 20%, the Israeli economy continued to suffer difficulties. In 1986, Israel replaced its currency with the new shekel (NIS). Even on a **global scale**, at the beginning of the decade, there was a severe global economic recession that significantly affected most developed countries. Later on, toward the end of the decade, the economy recovered due to the Laissez-faire policy (minimum governmental intervention in the economic affairs of individuals and society, thus, transactions between private parties are carried out freely) and the neoliberal economy in the developed world, led by the British and US governments, with an emphasis on reduced governmental intervention, tax reductions, and deregulation of the stock market. The success of Thatcher and Reagan's economic policies intensified the process of globalization; globalization is the expansion, acceleration, and deepening of cultural and economic ties between countries, societies, and individuals in a way that creates interaction and integration of economies, societies, cultures, and political movements all over the world. This situation led to interdependent relationships between companies, regardless of the nature of the connections.

Technological environment: During the decade, corporations such as IBM, Apple, and Commodore developed the PC (personal computer) for home use and Microsoft released a PC operating system. The accelerated development to build an internet network and the entry of the new system in 1989, the World Wide Web, enabled individuals to share information and link documents through an international network.

**B. S.W.O.T analysis:**

Strengths: The Druze community, following its traditional characteristics, is dominated by its traditional leadership; the Israeli establishment distinguishes the community from other minority sectors.

Weaknesses: The patriarchal and traditional structure of the Druze society imposes a close and conservative position on the development of other minorities.

Opportunities: The increase in the value of the community in the eyes of the Israeli establishment for participating and contributing to the state's security following the Druze martyrdoms during the intifada; the Lebanon War provided an opportunity for the (illegal) enrichment of individuals who served in Lebanon. Toward the end of the decade, it was apparent that the old traditional leadership was beginning to lose its status in favor of the younger generation.

Threats: The expansion of globalization in both social and economic terms and its penetration into Israel, marked the beginning of a change in the labor force in general and the employment of Druze women in particular. In addition, Western culture was spreading among the younger generation all over the country through global TV broadcasts, MTV channels, and more. The Druze community is beginning to face social and economic rifts.

**2. The ninth decade of the 20th century:**

**A. PEST analysis:**

Political: The end of the Cold War and the rise of the United States as a world superpower. China is beginning to stand out as an economic power, with the production of many consumer goods. During the Gulf War (January–February, 1991), Israel was attacked by Iraqi Scud missiles. In 1992, in the thirteenth elections to the Knesset (Israeli Parlament), the Labor Party, led by Yitzhak Rabin, won. The Oslo Accords were signed in 1993 and the peace agreement with Jordan in 1994. A lot of money was poured into improving the country's infrastructure, e.g., the building of many interchanges and highways. On November 4, 1995, Prime Minister Yitzhak Rabin was assassinated. The first Netanyahu government was in place from 1997 to 1999. The Barak government was in place from 1999 and 2001. The Four Mothers Movement increases the pressure on the government to withdraw IDF forces from Lebanon.

Economic environment: During the decade, the Israeli population grew from ~4.6 million inhabitants to ~6 million people due to the immigration of Jews from the former Soviet Union in the 1990s (the Jewish population increased from ~3.7 to ~4.9 million). Inflation continued to decrease. In addition, the policy of removing barriers in the capital market, free trade in foreign currency, the meteoric economic growth of the high-tech industry, especially the internet and telecommunications industries, made Israel a prominent global center in this industry. Although most of the hi-tech companies did not succeed in substantial financial terms, for several years they contributed to the inflow of foreign capital into Israel and supported certain sectors. The entry of international companies increased in the late 1980s, and as a result, foreign investment also increased significantly. At the same time, the Arab boycott weakened greatly. Israel has enjoyed high growth and relatively low unemployment in recent years. Under the auspices of globalization, production units were transferred to neighboring countries where labor is cheaper, mainly in the textile industry (more than 25 factories have copied their production units, e.g., Delta, Polgat, and Kitan).

Technology environment: The World Wide Web was launched in 1992 and by 1996 the internet had become widely distributed and businesses had begun to set up their home websites. By 1999, the internet had become widespread in every country in the world. Households in Israel began to consume PCs and cell phones had become popular in Western countries.

**B. SWOT analysis**:

Strengths: The community still maintained its traditional social characteristics (but the tendency was weakening). The State continued to distinguish between the community and other minorities. A young academic generation emerged that was highly aware of multiculturalism (due to military service and employment in various areas outside the village) and the traditional family leadership was replaced in the community. Many of the communities (villages such as Daliyat Al- Carmel, Usfiya, Yarka Julis, and Abu-Sanan) embraced the Western Israeli culture.

Weaknesses: Globalization reduced the development of the employment of Druze women in the textile industry, which was common in the villages. Israeli society was aimed toward business, women found themselves jobless, and the family abandoned the traditional source of income—agriculture. There was a transition from a traditional conservative patriarchal society to a modern one, with an adaptation of Western consumerism. The Druze community was divided between tradition and modernity.

Opportunities: The young generation realized the importance of higher education along with the authority's recognition of the accessibility of higher education in the periphery, which enabled young Druze women to acquire higher education, mainly in the field of teaching. Druze men enjoyed new employment opportunities in the private and public sectors outside the villages. Druze personal assets (illegal money) were invested in business, such as in the village of Yarka; these investments were a source of employment for both women and men in the villages. Druze men abandoned the field of teaching in favor of working in the private and public sectors outside their village (for higher salaries).

Threats: There was a decline in religious authority among the Druze youth (mainly after the death of Amin Tarif), an assimilation of Western culture, a weakening of the connection to the land (sailing lands, agriculture abandonment, etc.), a total dependence of Druze men on the Israeli (Jewish) labor market, a wide dependence on contract workers, and a decline in the traditional conservative identity of the community.

**3. The first decade of the 21st century**:

**A. PEST analysis:**

Political: IDF withdrawal from the Lebanese security zone (2000), an outbreak of the al-Aqsa Intifada (2000), Operation Defensive Shield (2002), the Second Lebanon War (2006), the implementation of the Israeli disengagement from Gaza (2005), and the Gaza War (2008). The precarious national security and the unstable economy brought back the “old politics”, which was oriented toward existential matters of the economy and security.

Economic environment: The outbreak of the Second Intifada led to a drastic decrease in foreign investment, the collapse of tourism, and severe damage to other industries. The global high-tech crisis—Israel, which during the 1990s established itself as a global center of start-ups, suffered the fall intensely. The crisis led to the downfall of hundreds of companies that were considered promising for the future of the State of Israel, and as a result, to major unemployment, and to an end of the “consuming fest” of the technological sector, which declined dramatically. In 2003, the “Economic Defensive Shield Program” was executed to reduce the public deficit. The development of free market policy, which brought back business, reduced government involvement and regulations, finally lead to a broad economic recovery, growth, reduced unemployment rates, the renewal of foreign investment, and minimal (almost zero) inflation. On the other hand, inequality increased significantly and the economic and social disparities increased the poverty indices.

The global economic crisis in 2008: The Bank of Israel took steps to lower interest rates to overcome the crisis. Despite these actions, inflation remained within a reasonable range (<4%), the unemployment rate was moderate (~8%) and then gradually returned to its pre-crisis level, GDP did not decrease, and foreign investment continued to flow into Israel. Employment—Despite the growth of the Israeli economy, the participation rate in the labor force (percentage of employees from the total labor force) was still relatively low at 56.3% compared with the average in developed economies, which was 70% in 2007. The main reason for the low participation rates in the labor force stemmed mainly from the absence of three sectors: 78% of Arab women, 65% of the strictly Ultra-Orthodox Jewish (Haredim) men, and 46% of the disabled.

Technological environment: Cell phones have become widespread across the world, as well as personal computers; the digital revolution has gone global.

**B. SWOT analysis**:

Strengths: The community still maintained its traditional core elements (although the tendency kept weakening). The Israeli establishment continued to differentiate the community from the other minorities. Young leadership was established; young Druze leaders manage to gain power within the Israeli Establishment (the Israeli parliament—the Knesset and senior command positions in the IDF and public service). The rise of the new religious leadership (grandson of Sheikh Amin Tarif, Sheikh Muwafak Tarif) took a liberal stand regarding women's employment and higher education. There was an increase in young Druze, especially women acquiring higher education. The Druze education system was identified with women. Druze men continue to work outside the village.

Weaknesses: The process of pounding the conservative Druze identity intensified. Druze youths embraced Western culture. The power of the extended family was greatly reduced while the status of the nuclear family was strengthened. There was an increase in the prestige of capitalists in the villages (usually through illegal activity) at the expense of the extended family; young Druze preferred easy money even at the cost of criminal activity. Religious authority continued to weaken and divorce rates increased. The identity crisis aggravated the Druze community, which was Socially and economically divided.

Opportunities: More colleges were established in the Galilee region, higher education became accessible, and the opportunities (fields of study) were great. Among Druze women, the tendency to study teaching intensified and the change was evident. In practice, there were more female teachers and female school principals. The IDF allowed Druze soldiers to join intelligence units that were closed to Druze soldiers in the past, an element that encouraged young Druze to join regular service. There was a continuation of Druze men tending to work outside the village in the Jewish labor market, especially if it involved large contracts. The development of private business in the villages accelerated. There was competition between Druze schools to increase achievement and the percentages of students eligible for a matriculation certificate.

Threats: There was a continued weakening of religious authority and a pounding of the conservative identity of the community. There was a spread of local leadership. Additionally, there was an expansion of the socio-economic gap between the Druze villages, as well as between individuals in the same village, both for reasons of globalization and due to the desire to be part of the Israeli establishment and a member of the ruling party.

**Figure 12:**
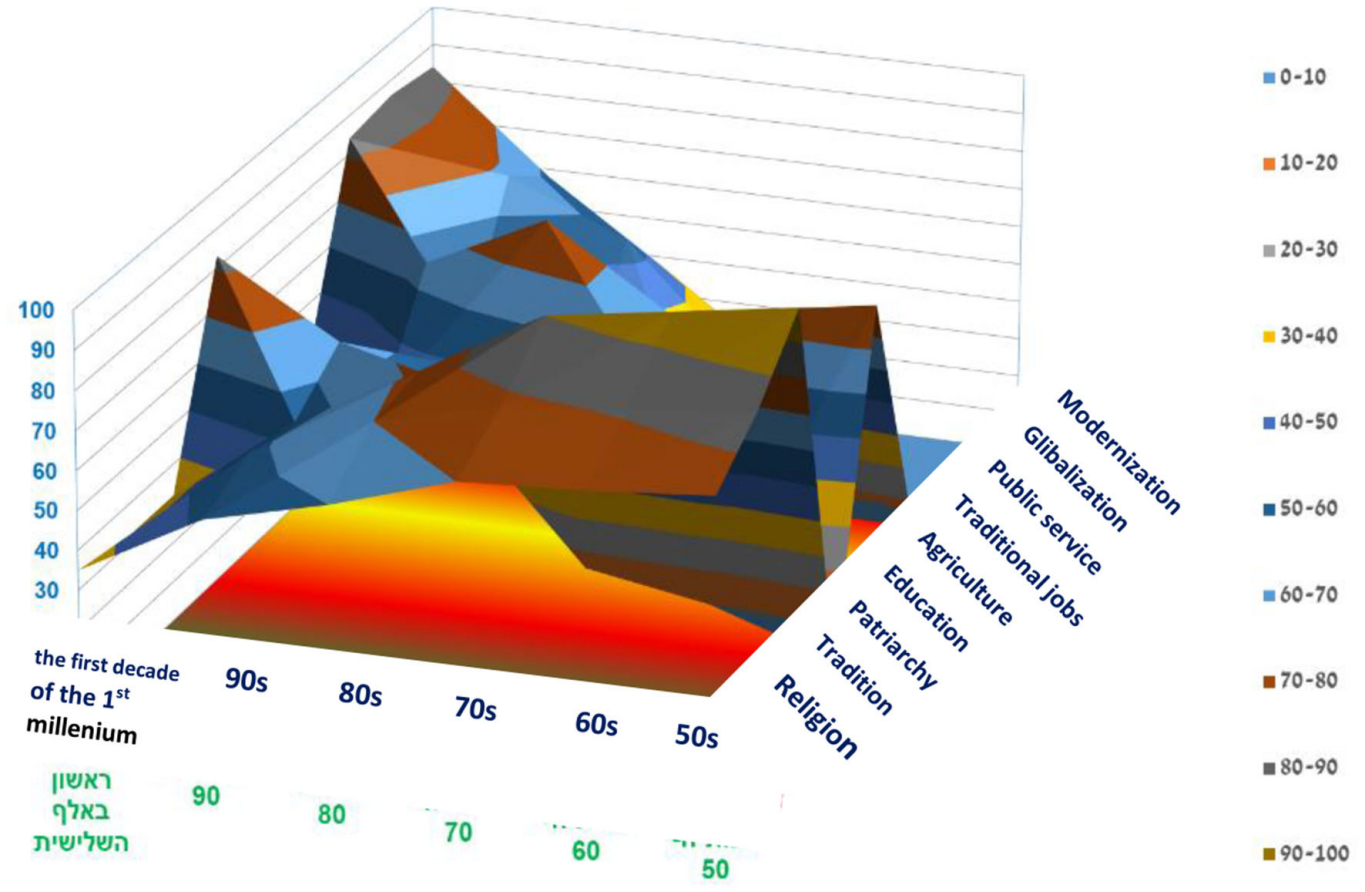
Surface diagram of the trends affecting the Druze community from the 1950s to the first decade of the third millennium (normalized) (self-cultivation).

The graph illustrates the trends that affected the Druze Community over 60 years since the establishment of the Israeli state, e.g., the diversion of tendencies during the 1980s and 1990s, such as the erosion of religion, tradition, patriarchy, the agricultural nature of work, and the reduction of traditional professions. At the same time, there was a trend of globalization and modernity, an increase in the field of academic education, and a transition to working in public services (education, health, etc.) (Figure 12).

#### 2.3.3. Analysis of the impact of the external environment on the internal environment in the last decade

This subsection analyzes the development of Druze women in the last decade (2010–2020), with a focus on labor and higher education.

**1. The second decade of the 21st century:**

**A. PEST analysis:**

Political: During the second decade of the 21st century, there was an unprecedented wave of uprisings, demonstrations, protests, and violence in the Arab world. In Israel, the second decade of the 21st century is mainly associated with the “Gilad Shalit Prisoner Exchange”, “Operation Amud Anan”, and “Operation Protective Edge” (Zuk Eitan). Throughout the decade, the ruling government was led by Benjamin Netanyahu. In 2018, the Nation-State Bill was enacted (Basic Law: Israel as the Nation-State of the Jewish People).

Economic environment: Israel moved to the biennial practice of budgeting. In 2011, there was a doctors' strike that lasted approximately four and a half months and became the longest strike in the history of the State of Israel. In the summer of 2011, the Israeli social justice protests opposed the continued increase in the cost of housing against the low average salaries between the years 2007 to 2011. The Trachtenberg Committee was appointed, and its recommendations were partially implemented, such as the Compulsory Education Law for kindergarten children and a permit to import food. From 2010 to 2016, the government raised taxes (corporate tax, national social security insurance payments, apartment tax, etc.). In the second decade of the 21st century, the economy in the state improved significantly. Thus, the growth rate was high (3–4% per year), as was the employment rate was also high regarding past salaries and in international terms and low GDP ratio. On the other hand, regarding labor productivity, the state is less effective and it is considered relatively low, for there are some sectors that integrate into the labor force partially (mainly Arab women and Orthodox Jewish men), in which inequality is high and the poverty rate is the highest among the Western countries.

Social environment: The social protest in Israel included a series of protests and demonstrations that took place throughout Israel in the summer of 2011. The protest expanded to include many other socio-economic issues besides housing. The Trachtenberg Committee was established, and later the government partially accepted the committee's recommendations. There were demonstrations during July against the Basic Law (The Jewish Nationality Bill). On August 4, 2018, the Druze leadership organized an assembly protesting the law and calling for equality. On August 11, 2018, a demonstration of the Supreme Monitoring Committee of the Israeli Arab Public was held in Rabin Square.

Technological environment: Cell phones have become as common as PCs. The digital revolution has become global, and technology is becoming dominant throughout the developed economies, as well as in Israel.

**B. SWOT analysis**:

Strengths: The process of the previous decade continued (maintaining the social core of traditional society). A liberal stance on employment and higher education was taken among young Druze women. The trend of acquiring higher education among young Druze is strengthened, especially among Druze women. The Druze education system acquired a female gender identity. Druze men continued to work outside the village. There is a growing trend among Druze women to become entrepreneurs and to join the business world; Druze women managed to break the glass ceiling. For the first time, a Druze judge was appointed, a Druze woman was elected to the Israeli parliament (the Knesset), and a Druze woman became an educational superintendent of Druze education. The first Druze surgeon in Israel was appointed. More Druze women studied medicine and acquired new professions, such as neuroscience; Ph.D. and more. For the first time, a Druze woman won The Israeli athletics championship, there was the first Druze police officer, the first Druze flight attendant, the first Druze basketball player, and the first Druze paramedic, etc.

Weaknesses: The establishment turned its back on the community with the enactment of the Nationality Law. There was a rise in young leadership. The new religious Leadership was facing opposition for supporting a reconciled approach toward the establishment. The process of identity pounding in the Druze conservative characteristics intensified and strengthened. There was an ongoing erosion of religious authority by the Druze youth. Druze youths embraced Western culture and capitalism strengthened. There was an increase in divorce rates, a religious identity crisis, and a shift toward modernity. The community was divided socially and economically.

Opportunities: More Druze women (especially teachers) were studying for a master's degree and Ph.D. The Druze education system became identified with and dominated by women. Many barriers were breached. The establishment recognized the great potential of Druze women.

Threats: There was a loss of conservative Druze identity. There were multiple local leaderships in Druze villages. The socio-economic gap widened and there was a return of the patriarchal voice.

## 3. Chapter 3. Discussion

**Figure 13:**
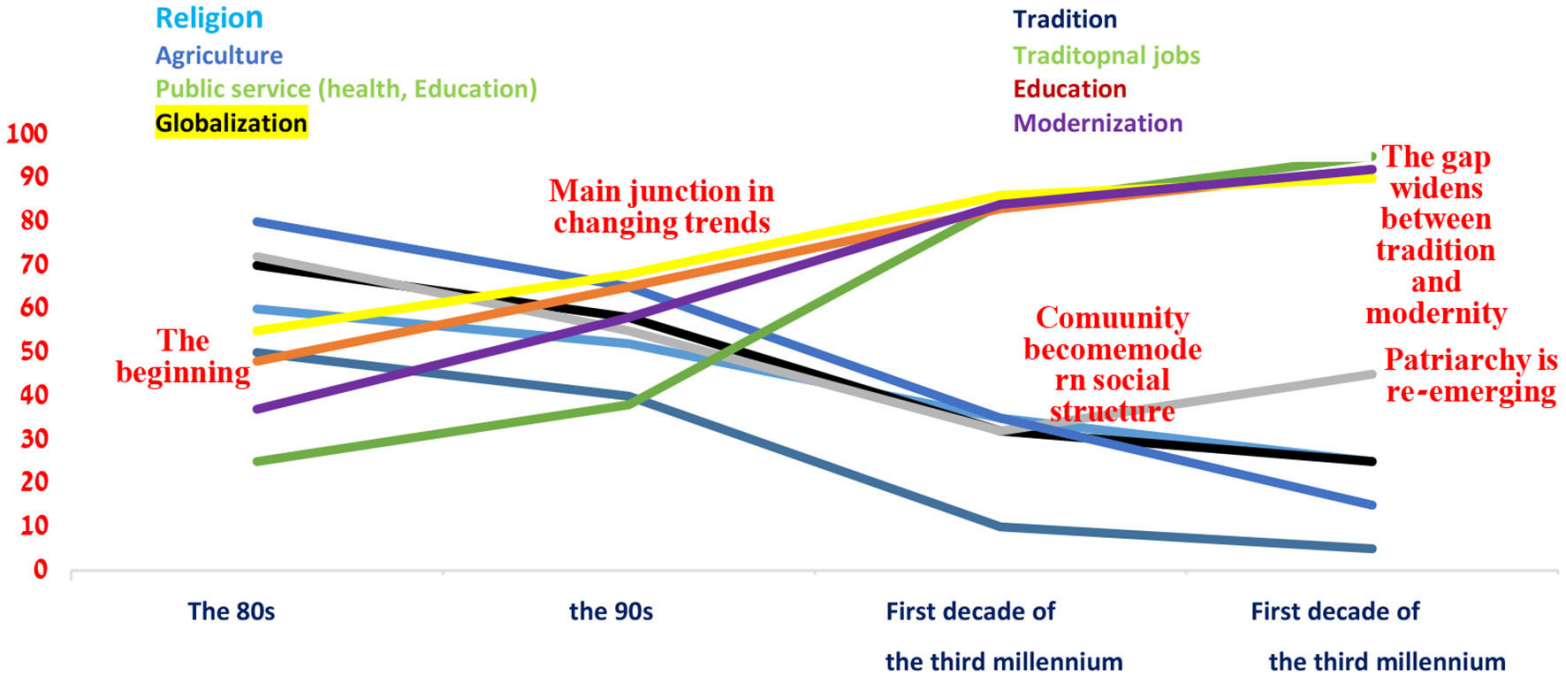
The trends affecting the Druze community from the 1980s to the second decade of the third millennium (self-cultivation).

This section presents empirical academic support for the above findings, as well as the specific factors that affected the development of Druze women in the last decade (Figure 13).

### 3.1. A discussion of the findings

This part reviews the implications of the findings. First, the findings prove that Druze women in the past and today suffer double discrimination; first, as a result of being part of a minority in Israel; and secondly, because they are to women in a traditional, rigid, and restrictive society. In the 1980s and 1990s, Druze women were employed in a limited number of industries and were reduced to certain professions in low-paying positions. The employed worked mainly in social media services and in the textile and food industries and small factories that were established by the Jewish entrepreneurs in the Druze settlements (Wiener Levi, 2004; Farraj Falah, 2006; Khatib, 2009; Yanai and Krauss, 2009; Amrani, 2010; Barkat, 2016).

In the early 1990s, it could be argued that the status of educated women in the family was inferior to men. Women were required to obey men, maintain a modest appearance, avoid contact with strangers, and take care of the housework and children (Farraj Falah et al., 2017). Moreover, for men, taking advice from a woman was considered shameful; therefore, men consulted other men in the clan.

The social and economic modification that took place in the Druze society benefited women in terms of education and labor; yet, it did not lead to normative modification toward democracy and equality due to opposition from the male hegemony and the advantages of the patriarchal order. The family mechanism, especially the dominant tribal clan in Arab society, is one of the most powerful when it comes to preserving the collective life pattern (A'li and Da'as, 2009; Karkabi-Sabah, 2009).

Globalization has many economic consequences all over the world and has a profound impact on the employment of Druze women. It has already been noted that the work of Druze women was limited to a small number of industries and professions, and as a result, their income was poor, both in the field of social community services and in industry, especially in textiles and food and small factories.

In the 1990s, globalization led to the shutdown of many factories, including in the field of textiles. Those industries were relocated to cheaper countries. For example, the number of jobs in the textile industry has decreased dramatically since 1990 from ~49,000 workers in the year 1990 to ~9,000 in the year 2017, a decrease of 82% (Izenkot, 2018). The main victims of this process were women; Arab women in general and Druze women in particular. As a result, women sought out for new employment and became contract workers, mainly in the public sector. They were not hired directly by the Government because of privatization in the public sector. The second channel was to acquire higher education to work as a teacher.

To locate the factors that led to the entry of Druze women into the labor force and to examine employment patterns, it is apparent that there is a difference between factors of supply and demand. The supply factors mainly include family and sociocultural elements, as well as factors related to changes in the life cycle, such as higher education—a factor with both direct and indirect effects, marriage patterns and family structure, childbirth, and previous experience working outside the home. Demand factors deal with motivation or a lack of motivation to work, including the income rate and the structure of the labor force (profession) in general and particularly in the peripheral Druze villages (Arar and Mustafa, 2009; Yashiv and Klinet-Kassir, 2018).

In the 1990s, higher education became available to other vulnerable populations, sectors that did not previously benefit from living in the periphery for instance; as a result, the proportion of students increased (Dagan-Buzaglo, 2007; Sheferman, 2007). The awareness of the importance of education along with the process of accessibility to institutions in the 1990s and 2000s had a great impact and modified the community's life in terms of education, the standard of living, consciousness, and ethnic recognition. From voluntary social isolation, the community began to open up to the world; if in the past (not too long ago), the vast majority of Druze youth devoted their activities to Druze society and its development, today, young Druze are concerned with self-fulfillment, studying at universities in Israel and around the world to acquire knowledge and improve their lives. During the last decade of the 20th century, young Druze began to embrace openness and independent thinking that encouraged doubting authority and tradition. The legitimacy of question and research did not stop at the level of study but also undermined the authoritative conception of absolute truth. Today, these personal and social developments allow the young Druze to gain respect in the community (Wiener Levi, 2005b; Kurt et al., 2012).

It is evident that the 1990s and the first decade of the 2000s constituted a major intersection in the structure of the community, both in the socio-economic aspect and in the aspect of identity. The Druze community has found itself existing in a dynamic environment driven by globalization. This kind of environment exerts external forces (economic, social, etc.) on the Druze Community, resulting in the closure of factories that employed Druze women. These external forces imparted a modern universal cultural perception that contradicted the existing traditional patriarchal structure of the community, which led to a social imbalance in the community's structure.

The 1990s became a platform for the growth of new modern concepts and drastic modifications in the community's structure. At the same time, the external environment expanded the offer of higher education, an element that had threatened the existing conservative traditional structure.

In the 1990s, Druze women were in a lower position compared with men. The inferior status of the Druze women within the family and society made it very difficult to obtain an education, especially when some villages did not have high schools within their territory. Despite this, approximately three decades ago, in the 1980s, Druze women who insisted on studying at university had to deal with conservatism and traditional closure, that is to say, the ban on leaving the village without a male escort, integrating with other sectors or sleeping outside the village. Very few families were willing to allow their daughters to study outside the village, and yet, over the years, girls from the various Druze villages began to attend the academy, breaking the traditional and social barriers, and today, a great number of Druze women attend universities and academic colleges in Israel (Farraj Falah, 2005; Barkat, 2016).

The recognition of the importance of academic education led to great changes in terms of the level of education, the standard of living, consciousness, and ethnic recognition. From voluntary social isolation, the community began to open up to the world outside. If in the past (not too long ago) the vast majority of Druze youth were dedicated to Druze society, these days, young Druze are concerned with personal development, learning, and earning money for themselves.

Over the past decade, the seeds of innovation, openness, and independent thinking were planted, enabling young Druze to question traditional authority. The legitimacy of questioning and investigating did not stop at the level of academic discourse; it persistently broke through the boundaries of the authoritative conception, which is no longer considered the absolute truth. This personal and social development paved the way for respect (Wiener Levi, 2005b; Kurt et al., 2012).

The appeal of Druze women to higher education changed their inferior status. Thus, the respected educated women serve as a role model for the entire community; Druze parents began to send their daughters to study so they could be respected by the community as well. These women, who challenged the pre-traditional norms paved the way for social change. Education enabled working outside the village, exposure to knowledge, a different way of thinking, other innovative approaches, and greater social involvement. This process created a “quiet revolution” that affected the social structure and status of Druze women. Those educated women returned to their villages and enabled change for Druze women in general. The nature of the “revolution” was not “Western feminist” against the “male world”; instead, the power of this change was in its invisibility, and therefore could not be suppressed. The aspiration of women to improve their status and break the oppression for the sake of other women challenged some traditional characteristics on the one hand and preserved others on the other hand (Farraj Falah, 2006).

In the 1990s, Druze women were victims of globalization as well as double structural discrimination, both from the Establishment, due to being part of the Druze minority, and simply due to being women in a traditional, rigid, and restrictive society.

Additionally, this pressure prevented women from working outside the village, mainly in the Arab peripheral rural areas that lack public transportation (Abu-Bader and Gottlieb, 2009; Yanai and Krauss, 2009). Furthermore, the dependence of the Druze economy on the Jewish economy limits employment opportunities for Arabs in general and Arab women in particular, and thus, Druze women are reluctant to work outside their hometown due to cultural-traditional motives. Khatib (2009) adds another dimension and argues that workplaces are “colorfully designed” by men, especially in the patriarchal Arab society in which men are the dominant, thus limiting women's access to higher positions. The employed minority among Druze women, work in a limited number of industries and occupations, in low-paid positions. As a result, women suffer from economic constraints and discrimination, both within and outside the family. The unequal employment structure of Palestinian society in Israel and the patriarchal social structure leave very few alternatives for women, educated or uneducated (Barkat, 2016). The result of the social and economic processes detailed above is evident in terms of today's employment distribution: the proportion of women working in agriculture is low and a higher proportion of women hold white-collar professions or semi-professional jobs (mainly as teachers and nurses); others are employed in the public service. Furthermore, the employment rate of women in manufacturing, as well as in factories owned by Jewish entrepreneurs in Druze localities, especially in the textile industry, has also increased (Fox and Friedman Wilson, 2018; Yashiv and Klinet-Kassir, 2018).

### 3.2. A discussion of the findings of the second decade of the 21st century

Druze women made great progress in the field of education, which had a great impact on employment, though it was more limited. The rate of Druze women with higher education increased, and employment rates are relatively high among women with an academic degree. These figures indicate that higher education provides opportunities for better employment rates and becomes a source of growth for the Israeli economy (Fox and Friedman Wilson, 2018).

Another interesting finding is that the percentage of female Druze students is higher than the percentage of male Druze students, a process that may eventually lead to a change in the community's social structure.

In the last decade, some trends could bring about change, and then the obvious question is, what are the factors that will lead to it? Why is the percentage of young Druze men applying to the academy smaller than the percentage of young women? What are the fields of study to which the academics applied? Why do they choose these specific fields? Is the academic integration of the Druze in the workforce equal to other sectors?

A careful examination of the findings proves that it is possible to assess the reasons that led to a sharp increase in the percentage of Druze women in the academy:

A. The Ministry of Education outlined a strategic plan to upgrade the level of teachers' education by opening a master's degree program for them.

B. Colleges offered master's degree programs to teachers. In terms of access to education in the periphery, the Ono Academic Campus began to operate master's degree programs for teachers; in 2015, Western Galilee College also began operating a special program, and in 2017, it opened a branch in Haifa City instead of the Carmel Academic Center. This process was dominant in the choice of Druze women teachers to acquire a master's degree.

C. The transition to a modern and even postmodern social structure resulted in changes in the pattern of marriage and the birth of Druze women. These changes enabled young Druze women first to acquire a higher education and only then to marry; after marriage and having a small family, the Druze woman is free to continue her academic studies. Moreover, Druze women began to study new professions other than teaching (as in the past), including professions that were considered masculine by traditional Druze society, such as medicine and engineering (Barkat, 2016).

D. The weakening of the religious authority and the rise of a young educated leadership enabled Druze women to acquire a higher education, without fearing a religious or social boycott by their families.

E. The recruitment policy of the State to IDF has changed since the beginning of the 2000s; Druze men could finally enter classified units that were previously closed to them, opening up new employment opportunities for young Druze women as Druze men began to abandon teaching and work outside the village. This situation was an opportunity for Druze women to replace men in these positions and integrate into teaching, thus meeting the community's expectations to maintain the Druze identity and preserve its values and norms.

F. The assimilation of Western consuming culture, which requires two spouses to work to make a living, motivated women to acquire higher education and work in the Ministry of Education. This tendency greatly assisted the process of feminization in the teaching profession.

G. Regarding employment, women seem to turn to work in customer service, such as call centers, sales centers, and outsourced support center companies.

Finally, it is necessary to take into consideration the emerging tendency of Druze women to seek careers in education, which is already a saturated field, and at the same time, the low percentage of women turning to science, high-tech, and engineering.

## Data availability statement

The original contributions presented in the study are included in the article/supplementary material, further inquiries can be directed to the corresponding author.

## Author contributions

The author confirms being the sole contributor of this work and has approved it for publication.

## References

[B1] Abu-Bader S. Gottlieb D. (2009). Poverty, Education and Employment in the Arab-Bedouin Society: A Comparative View. Jerusalem: National Insurance Institute, Research and Planning Administration.

[B2] A'li N. Da'as R. (2009). “The ideology of tasks and authority in the Palestinian family in Israel: gender inequality or trends of equality,” in Arab Women in Israel, Status Perspectives and Future, eds Azaiza F. Abu-Baker K. Hertz-Lazarowitz R. Ghanem A. (Tel Aviv: Ramot-Tel Aviv Universit), 25–45.

[B3] Amrani S. (2010). Between Community, Nation, and State. The Chaikin Chair for Geostrategy. Haifa: University of Haifa

[B4] Arar H. Mustafa M. (2009). “Higher education and employment among Arab women: barriers and changes,” in Arab Women in Israel –Status, Perspectives and Future, eds Azaiza F. Abu-Baker K. Hertz-Lazarowitz R. Ghanem A. (Tel Aviv: Tel Aviv University), 259–291.

[B5] Barkat A. (2016). Strategies and Practices of Druze Women Against Multiple Powers. Philosophy Ph.D. Essay. Ramat Gan: Department of Interdisciplinary Studies, Bar-Ilan University.

[B6] CBS (2015). Personnel Survey 2015. Jerusalem: Central Bureau of Statistics.

[B7] CBS (2021). The Druze Population in Israel - a Collection of Data on the Holiday of the Prophet Nabi Shuaib. Jerusalem: Central Bureau of Statistics.

[B8] Dagan-Buzaglo N. (2007). The Right to Acquire Higher Education in Israel: A Legal and Budgetary Perspective. Tel Aviv: Adva Center.

[B9] Falah S. (2000). The Druze in the Middle East. Tel Aviv: Adva Center.

[B10] Farraj Falah J. (2005). The Druze Woman. Tel Aviv: Barkai.

[B11] Farraj Falah J. (2006). Attitudes of druze youth in Israel toward druze women. Adult Educ. Isr. 9, 182–188.9199725

[B12] Farraj Falah J. (2016). The status of Druze women in the Druze religious law in comparison to Druze women's status in society. Int. J. Multidis. Res. Dev. 3, 203–206.

[B13] Farraj Falah J. (2017). “He is alienated”: intermarriage among druze men in Israel. Sociol. Mind 8, 70–82. 10.4236/sm.2018.81005

[B14] Farraj Falah J. Maman Y. Amasha W. (2017). Brides across the border (Syrian Druze bribes who have married Israeli Druze men of the golan heights after the Israeli occupation in 1967. Open J. Soc. Sci. 5, 289–303. 10.4236/jss.2017.55021

[B15] Fox H. Friedman Wilson T. (2018). Integration of Arab Women in the Labor Market: Education, Employment, and Wages. Jerusalem: Taub Center for Social Policy Studies in Israel.

[B16] Halabi Y. Shamai M. (2016). “The Israeli identity among druze adolescents in and the consequences of the violation of the civil rights of the arab minority on the identity,” in Conditional Citizenship: About Citizenship, Equality and Offensive Legislation, eds Uzetsky S. Jabarin Y. (Haifa: Pardes Publishing), 297–327.

[B17] Hassan Y. (2011). The Druze- Between Geography and Society, an Inside Look. Haifa: The Chaikin Chair for Geostrategy, University of Haifa.

[B18] Hazran Y. (2007). The Druze Community and the Lebanese State Between Confrontation and Reconciliation. Ph.D. Essay. Jerusalem: The Hebrew University.

[B19] Izenkot M. (2018). Description and Analysis of the Clothing and Textile Industries in Recent Decades. Jerusalem: The Knesset, Research and Information Center.

[B20] Johnson G. Scholes K. (2008). Exploring Corporate Strategy. Prentice-Hall, NJ: Pearson Education.

[B21] Karkabi-Sabah M. (2009). “The basis of the organization of the clan and the status of the arab woman” in Arab Women in Israel, Status Perspectives and Future, F. Azaiza, K. Abu-Baker, R. Hertz-Lazarowitz, and A. Ghanem (Ramot: Tel Aviv University), 47–70.

[B22] Khatib N. (2009). “It is not easy to be a Palestinian arab woman in Israel. Gender, ethnic and religious fines in the israeli workforce,” in Status and Future, eds Aziza F. Abu-Backer K. Hertz-Lazarowitz R. Ganes A. (Ramot: Tel Aviv University), 319–336.

[B23] Kurt D. Abbas R. Walters J. (2012). Identity Patterns and Educational Aspirations Among Druze Elders and Young Druze: Intergenerational Research on Attitudes and Perceptions. Ramat Gan: Bar-Ilan University.

[B24] Mezrahy S. H. Elyaho (2015). Collecting Data on Druze Womens Employment, Higher Education, and Intimate Partner Violence Against Women. Jerusalem: The Knesset, Research and Information Center.

[B25] Porter A. M. (1998). Competitive Strategy - Techniques for Analyzing Industries and Competitors. Israel: Ateret.

[B26] Samuel J. (2005). Organizations: Characteristics, Pattern and Processes. Haifa: University Press and Zmora Bitan.

[B27] Sheferman K. (2007). Inequality in Higher Education in Israel. Parliament 54. Jerusalem: The Israeli Institute for Democracy.

[B28] Shtrukman M. (2010). The Principle of Pluralism and Its Realization in the State of Israel. Tel Aviv: MATAH.

[B29] Veniger A. (2021). Data on Druze in the Higher Education System in Israel. Jerusalem: The Knesset, Research and Information Center.

[B30] Wiener Levi N. (2004). “*I am a Bird not yet Flying”. The Identity of the First Druze Women Who Gained Higher Education*. A Ph.D. Essay. Jerusalem: The Hebrew University of Jerusalem.

[B31] Wiener Levi N. (2005a). “Call me Umm al-Futa”: Higher Education as an Intercultural Encounter. Jerusalem: David Yellin College.

[B32] Wiener Levi N. (2005b). Higher education as a social and cultural encounter. Changes in the identity of pioneering druze women in acquiring higher education. social issues in Israel. J. Soc. Affairs. 1, 5–30.

[B33] Yanai Y. Krauss W. (2009). A Culture or a Structure of Opportunities: Why Do Palestinian Women Rarely Join the Work Force? Tel Aviv: Tel Aviv University.

[B34] Yashiv and Klinet-Kassir. (2018). The Economy of Arab Society in Israel. The Hebrew University; Am_Ovid Publisher.

